# The genetic relatedness of a peri-urban population of eastern grey kangaroos

**DOI:** 10.1186/s13104-018-3969-2

**Published:** 2018-12-04

**Authors:** Jai M. Green-Barber, Julie M. Old

**Affiliations:** School of Science and Health, Western Sydney University, Hawkesbury, Locked bag 1797, Penrith, NSW 2751 Australia

**Keywords:** Macropus, Microsatellite, Parentage, Sibship, Home range, Dispersal

## Abstract

**Objectives:**

The genetic diversity of an eastern grey kangaroo (*Macropus giganteus*) population surrounded by landscape barriers was examined. DNA was extracted from tissue samples from 22 road-killed kangaroos, and blood samples from four live captured kangaroos. Amplified loci were used to determine relatedness between individual kangaroos. The level of relatedness and location of road-killed kangaroos were compared to evaluate spatial autocorrelation.

**Results:**

The expected and observed heterozygosity confirmed the loci were polymorphic and highly informative for use in this population. One pair of kangaroos were identified to be full siblings, and a high proportion were identified as half siblings. Six positive parentage assignments were detected. No correlation between relatedness and crossing site was detected.

## Introduction

Social animals have distinct mating and dispersal patterns which influence the genetic diversity within populations [1]. Understanding how these mating and dispersal patterns influence genetic diversity is useful for developing management strategies and conservation efforts.

There is also insufficient data available regarding the genetic impacts of roads on macropods [2], and very little known about the group structure of eastern grey kangaroos (*Macropus giganteus*). Although there is some evidence that suggests that the social organization of eastern grey kangaroos is random, it may be more structured than previously thought as many studies have found non-random associations that relate to gender, age, and reproductive status [3–8]. Eastern grey kangaroo mobs (sub-populations) are comprised of multiple groups of long term associates with overlapping home ranges, that continually vary in size and composition [9]. The home range of male eastern grey kangaroos (7.6–269 ha) is often larger than that of females (9.3–248 ha), and males associate with multiple sets of females which vary in age and reproductive status [10–12]. Eastern grey kangaroos disperse randomly and do not appear to hold territories [13], however physical barriers in the landscape may limit movement, and low food availability causes populations to be more dispersed, hence both factors likely affect genetic diversity within a population [14]. The roads surrounding the site investigated in this study were identified as a road-kill hot spot for eastern grey kangaroos [15] and are likely to impact dispersal and gene flow of the study population.

Molecular techniques are commonly used to study populations [16], and relatedness of individuals determined using microsatellites [17]. Nine microsatellite markers were developed for eastern grey kangaroos by Zenger and Cooper [18], found to be highly polymorphic, and contain between eight and 19 alleles. The use of microsatellites will enhance and expand our knowledge of social, and spatial genetic structures in kangaroos, and can inform management strategies. This paper reports the genetic relationship of individuals in our study population described previously [15, 19]. Sibships and parent–offspring relationships were identified to determine whether there is a high level of relatedness in this population, and the correlation between relatedness and road crossing site examined. We hypothesise that due to the fragmented nature of the study site, there will be a high level of relatedness amongst the individuals sampled.

## Main text

Research was conducted at Yarramundi Paddocks (33°36′47.85″S/150°43′47.429″E), Western Sydney University’s (WSU) Hawkesbury campus as described previously [15, 19–21].

Ear clippings were collected from seven adult female, twelve adult male, and two juvenile (unknown sex) deceased kangaroos found on or near roads in the study area between July 2013 and October 2015. Body measurements, gender, GPS (Global Positioning System) location and general observations were also recorded [15]. Blood samples were taken from three live adult male kangaroos and one live juvenile male kangaroo following procedures described previously [19]. Genomic DNA was extracted from ear clippings and blood samples using the Qiagen DNeasy^®^ Blood and Tissue Kit (Qiagen, Hilden, Germany) following the manufacturer’s instructions.

Individuals were genotyped at 20 fluorescently labelled microsatellite loci using a primer panel compiled by Mark Eldridge. The panel included five tammar wallaby (*Macropus eugenii*) primers (Me) [22], since used for eastern grey kangaroos by Zenger, Eldridge and Cooper [23], five eastern grey kangaroo primers (G) [18], and seven primers isolated from the tammar wallaby and cross-amplified in the eastern grey kangaroo (T) [24]. Genotyping was carried out at the Australian Genome Research Facility, Melbourne.

Allele frequencies, PIC (Polymorphism Information Content), the observed heterozygosity (Ho) and expected heterozygosity (He), exclusion probabilities and estimate potential null-allele rates were calculated using CERVUS 3.0.7. GENEPOP 4.2 was used to test for departure from Hardy–Weinberg Equilibrium (p < 0.05) with 1000 iterations per 500 batches (S.E. (Standard Error) > 0.01), and genetic linkage disequilibrium (p < 0) with 1000 iterations per 1000 batches (S.E. > 0.01). Markov chain parameters were used for all tests and the log likelihood ratio statistic was also used for genetic linkage disequilibrium testing. Loci that did not deviate from the Hardy–Weinberg Equilibrium or have linkage disequilibrium, and had a null allele frequency < 0.05, were determined to be a potential marker to determine relatedness.

CERVUS 3.0.7 was used to determine the most likely parent pair for each candidate offspring using a pair-wise maximum likelihood estimation (LOD (Logarithm of the odds) > 0, mismatched loci ≤ 1). All kangaroos sampled were included as candidate offspring, however only adults were included as candidate parents. Full and half sibships were calculated (probability value ≥ 0.5) using COLONY 2.0.6.1.

The distance between pairs of road-killed kangaroos was measured using Google Earth and those found in close proximity were assumed to have overlapping home ranges. The majority of road-killed kangaroos were found on roads on the north-west side, and south-east side of the study site, and were assumed to utilize the adjacent semi-rural residential properties and Yarramundi Paddocks respectively. Linear regression (IBM SPSS Statistics 23) was used to evaluate the relationship (> 0.5) between the relatedness of pairs of road-killed kangaroos and the distance between their locations, and the direction of the road-kill from Yarramundi Paddocks.

Two adult male kangaroos were fitted with a colored ear tag (Allflex Pty Ltd, Capalaba Australia) and a VHF (very high frequency) tracking collar (Sirtrack Ltd, Hawkes Bay New Zealand). Kangaroos were tracked on foot from July 2014 to May 2016 and home ranges were estimated from data and analyzed using the online Zoa Track facility [25]. Locations were calculated using the GPS location of the researcher and the distance (determined by a range finder) of the kangaroo. Minimum convex polygons were calculated for all points (100%), approximate home range (95%), and core range (50%) based on the definitions of Jaremovic and Croft [26]. Polygons were calculated using the R package adehabitatHR within the ZoaTrack facility [27]. The proportion of overlapping ranges were calculated using the static territorial interaction equation as described in White and Garrott [28].

Microsatellite markers were amplified for 24 kangaroos. One locus (Me2) failed to amplify and was removed from further testing. Three loci (Me1, Me27, and T46.5) deviated from the Hardy–Weinberg Equilibrium and significant null allele frequencies were observed so were not included in further analysis. Significant null allele frequencies were observed for a further seven loci; however, these were within the Hardy–Weinberg Equilibrium and still included in analysis. Genetic linkage disequilibrium was not observed in any of the loci tested.

The mean He of microsatellite loci analyzed was 0.707, mean Ho 0.661, and mean PIC score 0.658 (Table 1), confirming the loci were polymorphic and highly informative. The number of alleles per locus ranged between 23 and 24 (mean 23.625). The majority (14/16) of the loci had He, Ho, and PIC scores >0.5, with the exception of loci G20.2 and T4.2. Previous studies have also reported heterozygosity estimates > 0.5 for other populations of eastern grey kangaroos [12, 23, 29].

**Table 1:** Genetic diversity estimates of 16 microsatellite loci used for kinship assignment of eastern grey kangaroos

Locus	N	Ho	He	PIC	F (null)	NE-1P	NE-2P	NE-PP
G15.4	23	0.870	0.848	0.808	− 0.027	0.512	0.340	0.165
G16.1	23	0.913	0.829	0.791	− 0.061	0.529	0.354	0.167
G20.2	24	0.417	0.508	0.469	0.080	0.864	0.701	0.523
G26-4	24	0.875	0.835	0.795	− 0.036	0.529	0.354	0.174
G31.1	23	0.696	0.708	0.653	− 0.005	0.713	0.534	0.342
Me14	24	0.625	0.786	0.734	0.111	0.623	0.445	0.262
Me15	24	0.542	0.674	0.619	0.106	0.747	0.570	0.379
Me16	23	0.696	0.807	0.759	0.061	0.587	0.408	0.224
Me17	23	0.696	0.839	0.796	0.082	0.534	0.359	0.182
Me28	24	0.708	0.795	0.750	0.048	0.593	0.414	0.223
T15.1	24	0.500	0.675	0.599	0.148	0.766	0.608	0.442
T19.1	23	0.870	0.700	0.659	− 0.185	0.701	0.512	0.301
T3.1T	24	0.708	0.704	0.648	− 0.020	0.719	0.541	0.352
T31.1	24	0.583	0.674	0.606	0.058	0.751	0.586	0.404
T32.1	24	0.667	0.734	0.672	0.038	0.692	0.520	0.336
T4.2	24	0.208	0.191	0.169	− 0.049	0.983	0.915	0.852
Mean	23.625	0.661	0.707	0.658	0.022	0.678	0.510	0.333

Number of alleles (N), observed heterozygosity (Ho), expected heterozygosity (He) polymorphism information content (PIC), the frequency of null alleles (F (null)), and average non-exclusion probabilities (NE-1P, NE-2P, NE-PP) as estimated by CERVUS

**Fig. 1:**
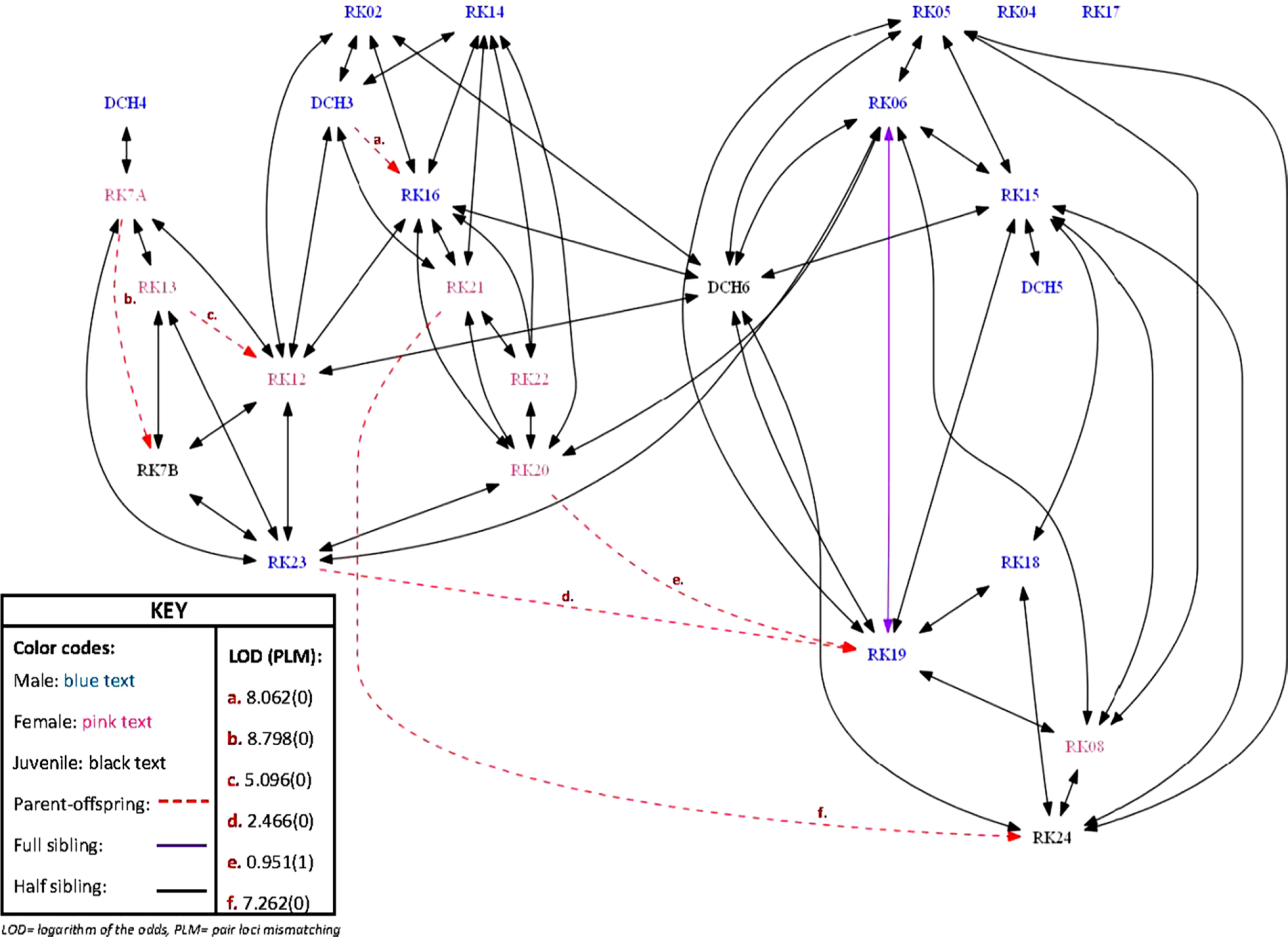
Genetic relationships of eastern grey kangaroos at Yarramundi Paddocks

Relationships were detected for 92% of kangaroos examined, however little evidence of genetic structure was observed which is consistent with previous research [12]. COLONY was used to assign sibship for 24 eastern grey kangaroos (Fig. 1). One full sibling was identified, and 54 half sibships were identified. Half of the identified sibships (50%) were between male and female kangaroos, and male–male sibships were more common (38%) than female–female sibships (12%). The average number of relationships was slightly higher for females (5.14) than males (4.29), and at least one relationship was detected for all female kangaroos sampled, however no relationships were detected for two male kangaroos (RK04 and RK17). Spatial genetic structure of eastern grey kangaroos is influenced by movement patterns, home ranges, and densities [30]. Males have larger home ranges and therefore disperse further than females [10], however the larger number of male–male sibships observed in this study may be an indication that dispersal is restricted, or may be a result of the greater number of males sampled in this study.

Parentage analysis was conducted for 21 candidate parents and 24 candidate offspring using CERVUS. Positive parentage assignments were determined for six offspring (Fig. 1). LOD scores ranged between 2.5 and 8.8. The majority (four) of the assigned parents were female and had positively identified offspring of both genders, whereas male parents only had positively identified male offspring. Kangaroos with offspring had a slightly higher average number of relationships (5.00 and 4.50 respectively) than other kangaroos in this study, indicating that individuals with more genetic relationships within the population also had greater reproductive success. In contrast Miller, Eldridge, Cooper and Herbert [29] found that female kangaroos prefer mates that are less genetically similar to avoid inbreeding. As it was not possible to sample all potential parents or offspring in our study population, it is likely that the parents and offspring of kangaroos sampled in this study were still present at the site.

Seventy-seven percent (N = 13) of kangaroos killed while crossing Londonderry road were related to other kangaroos killed on Londonderry road. No kangaroos killed while crossing Castlereagh road were related to other kangaroos killed on Castlereagh road, however 64% were related to kangaroos killed on Londonderry road and 36% were killed on other nearby roads. No significant correlation was found between the probability of relatedness and the distance between road-kill (R = 0.362) or the direction of road-kill from Yarramundi Paddocks (R = 0.314). Conversely, Neaves, Roberts, Herbert and Eldridge [12] reported a negative correlation between relatedness and geographic distance in eastern grey kangaroos. High levels of dispersal and immigration between populations is common in eastern grey kangaroos [23]. Our data is likely to be influenced by the majority of kangaroos dispersing in similar directions across Londonderry road to access resources at other sites, regardless of relatedness.

**Fig. 2:**
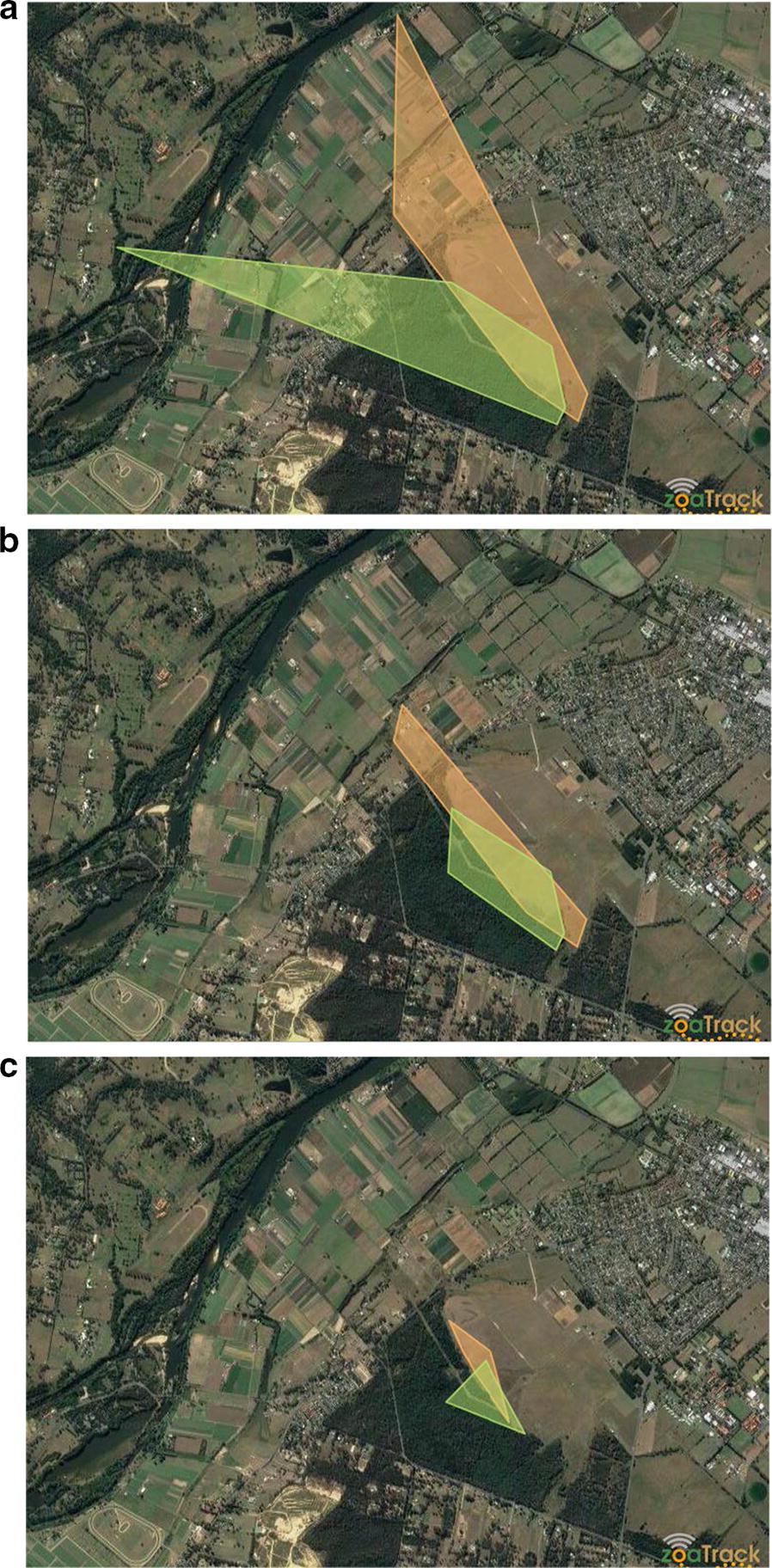
Home ranges of two adult male kangaroos at Yarramundi Paddocks made using Zoa Track [25, 27]. Key: *DCH3* orange, *DCH5* green. **a** All points (100%), **b** approximate home range (95%), **c** core range (50%)

The total home range of the collared male kangaroos was 170.9 ha (DCH3: 95% = 67.3 ha, 50% = 10.3 ha), and 174.8 ha (DCH5: 95% = 52.1 ha, 50% = 18.5 ha) (Fig. 2), and is consistent with previously reported home ranges of male eastern grey kangaroos [12]. Home ranges of both males included areas within Yarramundi Paddocks and the semi-rural residential properties to the north/west across Castlereagh road, however their approximate home ranges both occurred mostly within Yarramundi Paddocks, and their core ranges occurred entirely within Yarramundi Paddocks. The proportion of DCH3’s total home range was overlapped by DCH5 by 0.17 (95% = 0.42, 50% = 0.40), and the proportion of DCH5’s total home range overlapped by DCH3 was 0.16 (95% = 0.54, 50% = 0.22). These findings indicate that these kangaroos utilize a common area but occupy different ranges within the common area, and disperse in slightly different directions when travelling further from the core range. Sibship analysis determined a relatedness probability of 0.1 for half sibship of the two tracked males which does not constitute a positive sibship assignment. Unrelated male kangaroos may share overlapping home ranges because they utilize common resources such as grazing areas, resting spots, and mates. Adult male kangaroos reportedly associate with multiple females from different groups [10, 11], and the overlapping ranges within Yarramundi Paddocks may be indicative of the location of females within the overlapping range.

## Limitations

The sample size used in this study was limited by the availability of blood and tissue samples. Unfortunately, the kangaroos at the study site had a very large flight distance and were extremely difficult to capture to obtain blood samples, hence tissue samples were obtained opportunistically from road-killed kangaroos, which proved to be a more effective and non-invasive method of collecting samples for DNA extraction. The population density of kangaroos inhabiting the site was previously estimated as 4.6 kangaroos/ha [21], hence 1416 kangaroos were estimated to be utilizing the site. Based on the estimated population size at the site, this sample size accounts for less than 2% of the population, hence as this is a small sample of the larger population the high level of relatedness between individuals was not expected. The use of a greater number of individuals over a wider area, and more microsatellite markers is likely to have produced more robust results with reduced potential errors [31]. Despite limited sample size these findings are largely consistent with previous findings for this species and provides valuable insight into the genetic structure of this peri-urban population of eastern grey kangaroos which may assist managers of this population in the future.

## Abbreviations

GPS: Global Positioning System
Ho: observed heterozygosity
He: expected heterozygosity
LOD: logarithm of the odds
PIC: Polymorphism Information Content
S.E.: Standard Error
VHF: very high frequency
